# The return of Chinese nurses from overseas (2009–2023): a mixed-method study on influencing factors

**DOI:** 10.1186/s12992-025-01157-w

**Published:** 2025-11-03

**Authors:** Xinyi Liao, Shuting Li, Mengqi Li, Jun Wang, Jushuang Li, Huaping Liu, Ling Wang, Lili Fu, Chunli Zhang, Mengdie Li, Lina Yi, Chun Hao

**Affiliations:** 1 Department of Medical Statistics and Epidemiology, School of Public Health, Sun Yat-Sen University, No. 74, Zhongshan Second Road, Guangzhou, Guangdong 510080 P. R. China; 2 Sun Yat-Sen Global Health Institute, Institute of State Governance, Sun Yat-Sen University, Guangzhou, Guangdong P. R. China; 3 Health Human Resource Development Center, National Health Commission of the People’s Republic of China, No. 3, Huoqiying Road, Beijing, 100080 P. R. China; 4 School of Nursing, The Hong Kong Polytechnic University, Hong Kong, P. R. China; 5 School of Nursing, Chinese Academy of Medical Sciences & Peking Union Medical College, Beijing, P. R. China; 6 Nursing Department, Peking University People’s Hospital, Beijing, P. R. China

**Keywords:** International nurse, Mixed methods, Return migration, Job opportunity, Health professional, China

## Abstract

**Background:**

The shortage of nurses poses a growing threat to global healthcare quality, particularly in low- and middle-income countries. Out-flow of nurses from these regions to developed countries exacerbates the global imbalance in nursing resources. However, some nurses decide to return home after gaining experience abroad. Understanding their motivations, challenges, and opportunities can offer valuable insights for China and other nations facing similar challenges.

**Methods:**

Data were collected from October to November 2023, with a mixed-methods research design. Trends and the distribution of returning nurses were visualized. Cox proportional hazards regression and subgroup analyses were used to examine the relationship between associated factors and return rate. Qualitative data, gathered through semi-structure interview, were analyzed using inductive thematic analysis.

**Results:**

29.69% participants have returned to China, with the cumulative incidence of return was 47.01% (95% CI 37.96%-57.04%) after 13 years, varying by destination. In the multivariable Cox model, factors like age 30–39 (adjusted HR [aHR] = 0.48, 95%CI 0.31–0.74), advanced language proficiency (aHR = 0.30, 95%CI 0.14–0.64), and average annual income abroad of $50,000: $100,000 (aHR = 0.32, 95%CI 0.20–0.52) and over $100,000 (aHR = 0.52, 95%CI = 0.33–0.83) were independently associated with return rates. Interviews with returning nurses revealed three main themes: (1) challenges faced overseas, mainly language barriers; (2) reasons for return, particularly family considerations; and (3) challenges and opportunities after returning, with better chance in private hospitals as a notable subtheme.

**Conclusions:**

This study provides the first comprehensive analysis of Chinese nurses returning from abroad, revealing that nearly 30% returned, mainly due to family considerations. More skilled nurses, with international licenses and higher incomes, tend to stay abroad. Most of these returning nurses secure employment in private hospitals, while reintegration into public hospitals proves challenging. The study indicates the potential to leverage returning nurses’ global expertise to enhance China’s healthcare system.

**Supplementary Information:**

The online version contains supplementary material available at 10.1186/s12992-025-01157-w.

## Background

Nurses play a crucial role in healthcare, engaging in health promotion, disease prevention, and direct patient care [1]. According to the World Health Organization’s “State of the World’s Nursing 2020,” nurses make up about 59% of healthcare professionals globally [2]. However, a significant nurse shortage of over 5 million exists, with 89% of this gap in low- and middle-income countries [2]. One major contributor to this shortage is nurse migration [3, 4], as many nurses from these countries seek better opportunities abroad [5, 6], posing retention challenges for nations like China [7, 8].

Compared to developed countries, China’s nursing workforce is markedly lacking [8]. In 2020, the nurse-to-population ratio in China was 3.3 per 1,000 people [9], while countries like the U.S., Australia, and Singapore, the most popular destination countries for Chinese migrant nurses [10], had ratios of 12.5, 16.4, and 7.4, respectively [9]. Additionally, there is a notable shortage of experienced nurses in China, with only 41.4% having more than ten years of experience by 2022, compared to 69% in the United States [11, 12]. Moreover, over 70% of registered nurses in the United States hold a bachelor’s degree or higher, while only 30.4% do in China [11, 12]. Nonetheless, many skilled Chinese nurses move to countries with better nursing resources [13], resulting in a “brain drain” that worsens this disparity [14].

Despite the outflow, emigration is not always permanent. The International Center for Nurse Migration reports that about 50% of skilled health workers return home after time abroad [15]. This trend reflects a shift from “brain drain” to “brain circulation” [16], where returning professionals bring valuable skills and knowledge acquired overseas [17]. Nurses with international experience enhance their language proficiency and contribute expertise across various domains. Their return can significantly enrich the nursing workforce, impacting management roles and mentorship for future nurses [18].

There is very limited data on nurse return migration, with only a few studies reporting return rates [15]. For example, research from Romania indicates a return rate of about 31%, while in the Philippines, the rate is around 18% [19, 20]. However, no data on nurse return rates has been reported for China. Additionally, some studies have explored factors influencing nurse return migration. For instance, studies using the push-pull theory [21, 22] found that economic incentives in the home country often motivate nurses to return, while push factors from host countries, such as integration difficulties, tend to have less impact. However, most of these studies are qualitative. Moreover, factors influencing return migration differ significantly across countries due to cultural variations [23], and no relevant studies have been r conducted on China yet.

This study utilizes a mixed-methods approach to examine the factors affecting Chinese registered nurses who have worked abroad and returned to China, and to explore the challenges and opportunities they faced. Understanding these elements can guide healthcare policies, address workforce shortages, and optimize nursing talent deployment to China and other countries facing similar challenges.

## Methods

### Study design and data collection

This study adopted a two-phase explanatory sequential mixed-methods design. In the first phase, a questionnaire survey was administered to Chinese nurses with overseas experience. In the second phase, one-to-one interviews were conducted with a subset of returnee nurses. The quantitative survey provided an overall understanding of the research problem, while the qualitative interviews offered deeper insights into factors requiring further clarification [24].

The email-based survey targeted all nurse applicants listed in China’s National Credentials Verification Center for Health Professional. Established by the National Health Commission in 2007, the center is the only official institution in China that certifies health professional’s domestic practice credentials, meeting their needs to work or study abroad. From the implementation of the digital system on September 30, 2009, to August 31, 2023, 6,583 registered nurses applied for credential verification. Applicants were told at signup that emails might be used for follow-up surveys and research.

A survey was conducted from October to November 2023, targeting 6,583 nurse applicants via email. Invitations were sent to those with a valid email address who had previously agreed to be contacted, each containing a brief outline of the study and a link to the questionnaire. Follow-up reminder emails were sent weekly, for a total of four contact attempts to non-respondents. Individuals who did not respond after these attempts were considered to have declined participation.

The analysis included only nurses who had worked or lived abroad after obtaining verification from the Credentials Verification Center for Health Professionals and who provided consent. Applicants from other professions, such as physicians, were excluded.

To explore reasons for return and challenges faced by nurses, telephone interviews were conducted from January to February 2024, targeting survey respondents who have returned to China. The interviews were conducted in Chinese by author XL, while author SL, responsible for verifying the participants’ professional credentials, recorded the entire process and translated it into English. Each interview lasted approximately 20 min, and the audio recordings were transcribed and checked for accuracy. Participants were subsequently invited to confirm the accuracy of the transcriptions. Both the original transcripts and the synthesized findings were reviewed by nursing experts HL and LW.

Participants were purposively selected to ensure diversity in professional and personal backgrounds, considering factors such as duration of stay overseas, possession of nursing practice licenses abroad, income, and language proficiency. Recruitment continued until data saturation was reached, i.e., when no new subthemes emerged.

The mixed-method study received ethical approval from Sun Yat-sen University (SYSU-SPH149) for research involving human participants. Non-public data, including email addresses, demographic information, and application records, were obtained with explicit authorization from the Credentials Verification Center for Health Professionals, and only the permitted data were used in this study. Informed consent was obtained from all participants prior to the survey and interviews, and they were informed of the study’s purpose. Participation was voluntary, and confidentiality was strictly maintained, with access to the data restricted to the research team.

### Measurements

The online questionnaire was developed based on a literature review [19, 25–27] and guided by informed by Young’s model of migration, focusing on meso-level (profession-led) and micro-level (personal) drivers [27, 28]. Then, it was reviewed by experts in nursing, health policy, and health workforce, and piloted with eight emigrant nurses who were excluded from the final analysis. It covered three sections: demographic characteristics, work-related information, and international mobility status [see Additional file 1].

i). **Demographic Characteristics**: Included variables such as age, education, marital status, and parenthood.
ii). **Work-Related Information**: This included both domestic and overseas employment, covering the workplace province before going abroad, type of employing institution (in line with the *China’s Health Statistical Yearbook 2022* [12], including hospitals—public and private, primary healthcare institutions, public health institutions, and other institutions), nursing practice level within the Chinese health system (assessed through the national nurse professional evaluation: senior- chief nurse/associate chief nurse; middle-supervisor nurse; entry-nurse), self-assessed language proficiency before going abroad (categorized as basic, intermediate, or advanced), average annual income abroad, whether possess a full legal nursing license in the host country, and whether the respondent continued working in the nursing profession after returning to China (including clinical care, education, healthcare enterprises, or other related sectors).
iii). **International** **Mobility**: Included destination country, the year they left China, primary purpose for going abroad (study, employment/migration), return status, year of return, current workplace (province) if still engaged in the nursing profession, and reasons for returning as interpreted through migration theory.

The semi-structured guide was based on previous literature and qualitative data and reviewed by experts, focusing on decision factors for going abroad and returning, as well as abroad and post-return work situations to gain deeper insights into participants’ experiences [see Additional file 2].

### Data analysis

#### Quantitative Analysis 

Demographic characteristics between respondents, non-respondents (valid emails) and nurses without valid email addresses were compared using Chi-square, Fisher’s exact, or analysis of variance (ANOVA), with post-hoc pairwise comparisons conducted using the Bonferroni correction. Descriptive analysis was used for categorical variables, presented as counts and percentages, and baseline variables were compared between nurses who returned and those who did not using the Chi-square method. The primary outcome, “returned to China” was analyzed using the duration abroad as the years at risk. Mann-Kendall test analyzed return rate trends, and heat maps depicted return rates by destination and domestic workplace. Kaplan-Meier method generated the cumulative incidence curve for return. Univariate and Cox multivariate regression models explored associations with outcomes, controlling for confounders. Subgroup analysis considered different purposes for going abroad. The assumption of proportional hazards was examined by a Schoenfeld test. All analyses and graphs were conducted in RStudio (version 1.4.1103). Significance was set at *P* < 0.05.

#### Qualitative Analysis

Interview data were transcribed verbatim by two investigators and analyzed thematically with the help of QSR NVIVO 12 software [29]. Two investigators independently coded the transcripts, creating descriptive codes that were subsequently grouped into subthemes and major themes through discussion and consensus. Representative quotations were extracted verbatim to support the subthemes.

## Results

### Participant characteristics

**Fig. 1:**
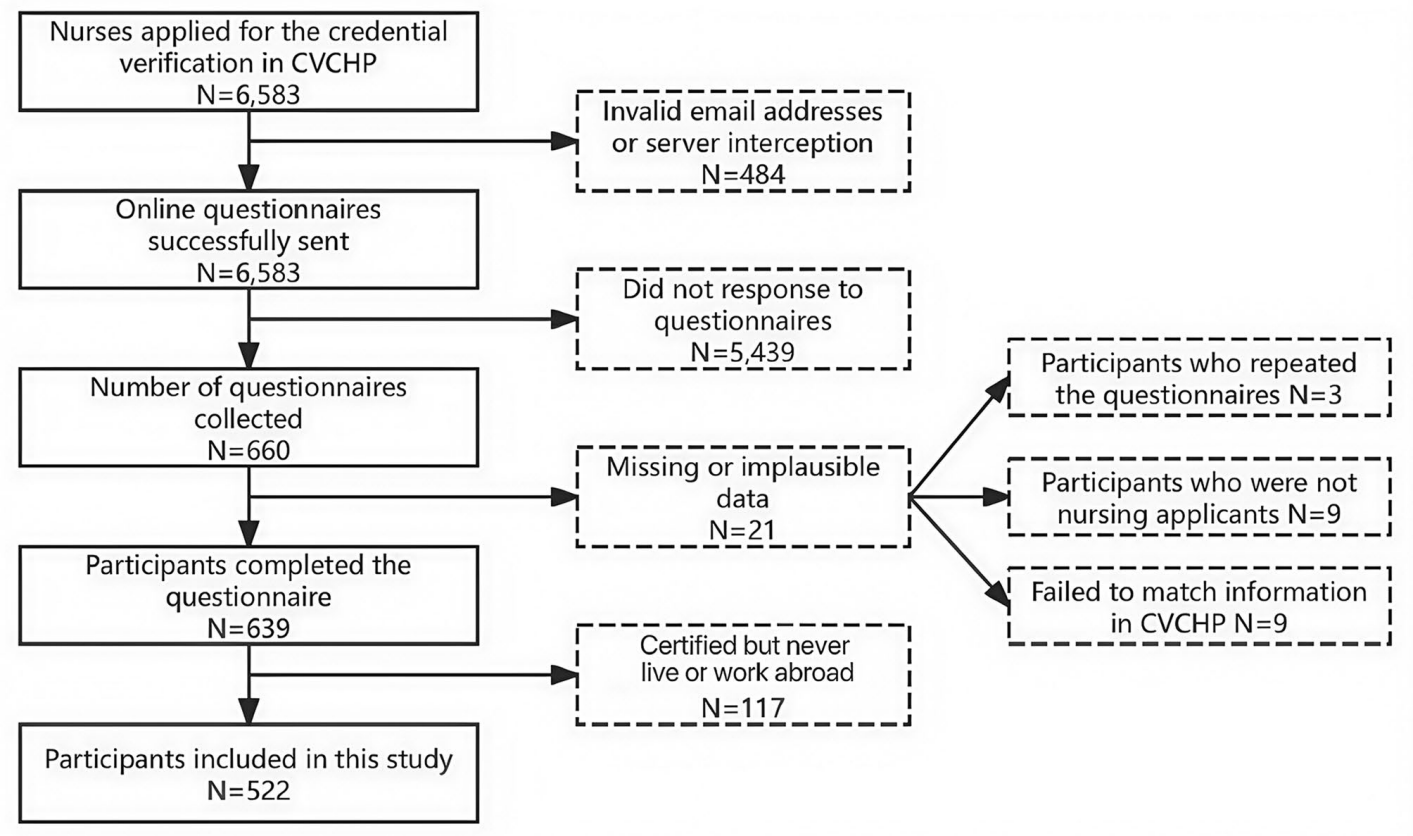
Flowchart of the inclusion procedure

The study population consisted of 6,583 nurse applicants to the Credentials Verification Center for Health Professional between September 30, 2009, and August 31, 2023. Of the 6,583 emails sent, 92.6% (6,099) were successfully delivered, with a response rate of 10.82% (660). Among respondents, 96.82% (639) completed the questionnaires and were identifiable in the Credentials Verification Center for Health Professional, with 81.69% (522) being eligible for the survey (Fig. 1). Demographic and profession-related comparisons of participants, non-respondents, and nurses without valid email addresses are provided in Additional File 3. Respondents had a significantly later application year than both non-respondents with valid emails and nurses without valid emails, as well as a significantly higher educational level. They were also younger than nurses without valid email addresses.

As of August 31, 2023, 155 of 522 overseas nurses had returned to China (29.7% return rate). Table 1 compares characteristics of returnees and non-returnees. Younger, unmarried nurses and those without children were more likely to return, while those with a bachelor’s degree or higher, greater language proficiency, or annual incomes above $50,000 were less likely to return. Nurses who went abroad for study were more likely to return than those who went for work or migration.

**Table 1:** Associations between baseline characteristics and nurse return rates (univariate analysis)

Variable	Total (%) (*N* = 522)	Return (raw%) (*n* = 155)	OR	*P*
Age				
29 and below	217 (41.57)	98 (45.16)	1.00 (Reference)	-
30–39	221 (42.34)	40 (18.10)	0.27 (0.17–0.42)	< 0.001
40 and above	84 (16.09)	17 (20.24)	0.31 (0.17–0.56)	< 0.001
Education				
Post-secondary or below	188 (36.02)	76 (40.43)	1.00 (Reference)	-
Bachelor or above	334 (63.98)	79 (23.65)	0.46 (0.31–0.67)	< 0.001
Marital status				
Unmarried	204 (39.08)	75 (36.76)	1.00 (Reference)	-
Married	318 (60.92)	80 (25.16)	0.58 (0.39–0.85)	0.006
Parenthood status				
Having children	307 (58.81)	76 (24.76)	1.00 (Reference)	-
Childless	215 (41.19)	79 (36.74)	1.77 (1.21–2.58)	0.004
Domestic employment institution				
Hospital	341 (65.33)	107 (31.38)	1.00 (Reference)	-
Public hospital	251 (48.08)	-	-	-
Private hospital	90 (17.24)	-	-	-
Primary healthcare clinic	33 (6.32)	11 (33.33)	1.09 (0.51–2.33)	0.845
Public health institution	14 (2.68)	1 (7.14)	0.17 (0.02–1.33)	0.073
Other	134 (25.67)	36 (26.87)	0.80 (0.51–1.25)	0.375
Level of nursing practice				
Entry-level	448 (85.82)	138 (30.80)	1.00 (Reference)	-
Middle level or above	74 (14.18)	17 (22.97)	0.67 (0.38–1.19)	0.216
Language proficiency				
Basic	76 (14.56)	35 (46.05)	1.00 (Reference)	-
Intermediate	329 (63.03)	106 (32.22)	0.56 (0.34–0.92)	0.023
Advanced	117 (22.41)	14 (11.97)	0.16 (0.08–0.33)	< 0.001
Purpose of going abroad				
Study	60 (11.49)	29 (48.33)	1.00 (Reference)	-
Work or migrant	462 (88.51)	126 (27.27)	0.40 (0.23–0.69)	< 0.001
Whether obtain nursing practice licenses abroad				
Yes	397 (76.05)	110 (27.71)	0.68 (0.45–1.03)	0.092
No	125 (23.95)	45 (36.00)	1.00 (Reference)	-
Average annual income abroad				
Below $50,000	176 (33.72)	88 (50.00)	1.00 (Reference)	-
$50,000: $100,000	199 (38.12)	34 (17.09)	0.21 (0.13–0.33)	< 0.001
Over $100,000	147 (28.16)	33 (22.45)	0.29 (0.18–0.47)	< 0.001

The semi-structured interviews involved 7 participants, all of whom had returned to China. The participants had a mean age of 39 years (SD = 7.06); 6 held a bachelor’s degree or higher. 5 participants had stayed overseas for ≤ 3 years, and 5 had obtained nursing practice licenses in their destination countries. Participants returned from a range of countries: 2 from the United States, 2 from Singapore, 2 from Saudi Arabia, and 1 from the United Kingdom. The thematic analysis generated 3 main themes, and 12 subthemes.

### Distribution of overseas and returned Chinese nurses

**Fig. 2:**
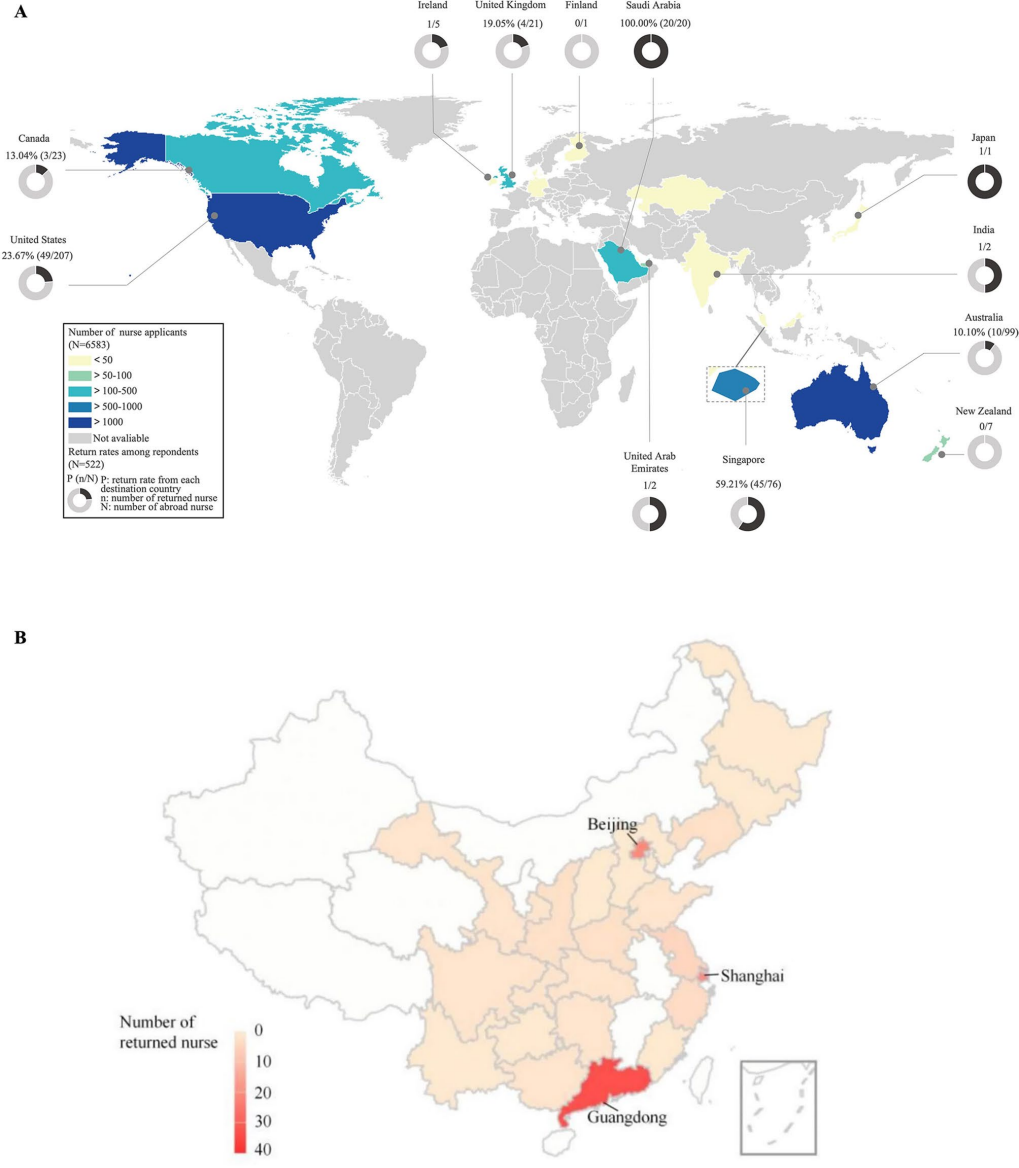
The number and return rates of Chinese nurses by destination countries (**A**) and distribution of the 120 nurses continuing nursing career after returning (**B**)

According to the Credentials Verification Center for Health Professional records, the top three destination countries for nurse credentials verification from 2009 to 2023 were the United States, Australia, and Singapore [see Additional file 4]. Return rates in these three countries were 23.67%, 10.10% and 59.21%, respectively. Asian countries showed the highest return rates, reaching 100% in Saudi Arabia, with other Asian nations also showing rates of 50% or higher. By contrast, return rates in American, European and Oceania countries were no more than 25% (Fig. 2A).

120 out of 155 (77.42%) returning nurses continued their nursing careers in China, and the majority practiced in the provinces and municipalities of Guangdong, Beijing, and Shanghai, with respective proportions of 26.67% (*n* = 32), 17.50% (*n* = 21), and 16.67% (*n* = 20) (Fig. 2B).

### Return rate over the period from 2009 to 2023

**Fig. 3:**
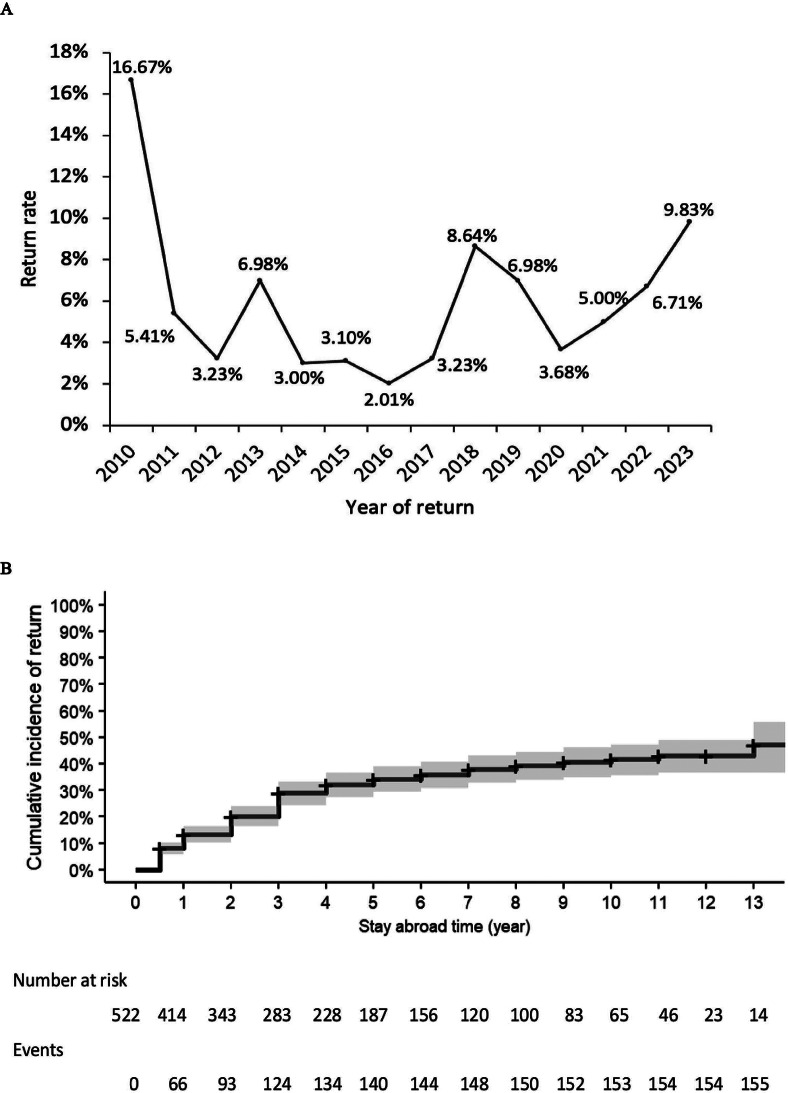
The trend of return rate over years from 2010 to 2023 (**A**) and Kaplan-Meier cumulative incidence curve for return (**B**)

The return rate fluctuated significantly over the years without clear trends (*P* = 0.583), peaking at 16.67% in 2010 before declining to 2.01% in 2016. From 2020 to 2023, it rose from 3.68% to 9.83% (Fig. 3A). The estimated cumulative incidence of return was 28.94% (95%CI 24.79%-33.61%) after 3 years and 47.01% (95%CI 37.96%-57.04%) after 13 years (Fig. 3B).

### Cox proportional hazards analysis of factors influencing nurse return

Between 2009 and 2023, 155 out of 522 participants have returned (7.31 per 100 person years, 95CI% 6.25–8.52). Univariate analyses were guided by Young’s migration model, and variables significant in univariate analyses (*P* < 0.05) were included in the multivariate Cox model. The multivariable Cox model (Table 2) identified several factors independently associated with return: being aged 30–39 (adjusted HR [aHR] = 0.55, 95%CI 0.37–0.82), advanced language proficiency (aHR = 0.38, 95%CI 0.19–0.74), going abroad for work or migration (aHR = 0.52, 95%CI 0.34–0.80), higher annual income of $50,000: $100,000 (aHR = 0.33, 95%CI 0.22–0.50) or over $100,000 (aHR = 0.52, 95%CI = 0.34–0.80), and obtaining nursing practice license abroad (aHR = 0.62, 95%CI = 0.43–0.90).

**Table 2:** Risk of return according to timing and characteristics

Variable	No of return	Incidence rate per 100 person years (95%CI)	Unadjusted HR (95%CI)	Adjusted HR (95%CI)
All	155	7.31 (6.25–8.52)		
Age				
29 and below	98	9.55 (7.86–11.56)	1 (reference)	1 (reference)
30–39	40	4.59 (3.34–6.25)	0.45 (0.31–0.65)^***^	0.55 (0.37–0.82)^**^
40 and above	17	7.61 (4.63–12.11)	0.67 (0.40–1.13)	0.91 (0.51–1.62)
Education				
Post-secondary or below	76	9.85 (7.88–12.23)	1 (reference)	1 (reference)
Bachelor or above	79	5.85 (4.69–7.28)	0.60 (0.44–0.82)^**^	0.87 (0.61–1.25)
Marital status				
Unmarried	75	10.47 (8.38–13.01)	1 (reference)	1 (reference)
Married	80	5.69 (4.57–7.07)	0.61 (0.44–0.84)^**^	0.88 (0.56–1.37)
Parenthood status				
Childless	79	10.85 (8.73–13.39)	1 (reference)	1 (reference)
With children	76	5.46 (4.35–6.82)	0.58 (0.42–0.80)^**^	0.89 (0.55–1.44)
Domestic employment organization				
Other	36	7.62 (5.46–10.49)	1 (reference)	-
Hospital	107	7.21 (5.97–8.67)	1.05 (0.72–1.54)	-
Primary healthcare clinic	11	9.52 (5.08–16.77)	1.29 (0.66–2.53)	-
Public health institution	1	2.08 (0.11–12.47)	0.27 (0.04–1.93)	-
Level of nursing practice				
Entry-level	138	7.52 (6.37–8.84)	1 (reference)	-
Middle level or above	17	5.95 (3.61–9.54)	0.82 (0.49–1.36)	-
Language proficiency				
Basic	35	12.87 (9.24–17.57)	1 (reference)	1 (reference)
Intermediate	106	8.15 (6.75–9.8)	0.66 (0.45–0.97)^*^	0.85 (0.56–1.29)
Advanced	14	2.55 (1.46–4.35)	0.21 (0.12–0.40)^***^	0.38 (0.19–0.74)^**^
Purpose of going abroad				
Study	29	13.91(9.66–19.53)	1 (reference)	1 (reference)
Work/migration	126	6.59 (5.54–7.82)	0.48 (0.32–0.72)^***^	0.52 (0.34–0.80)^**^
Average annual income abroad				
Below $50,000	88	16.81 (13.76–20.35)	1 (reference)	1 (reference)
$50,000-$100,000	34	3.27 (2.31–4.59)	0.23 (0.15–0.34)^***^	0.33 (0.22–0.50)^***^
Over $100,000	33	5.2 (4.17–8.3)	0.39 (0.26–0.58)^***^	0.52 (0.34–0.80)^**^
Whether obtain nursing practice licenses abroad				
No	45	16.27 (12.23–21.28)	1 (reference)	1 (reference)
Yes	110	5.96 (4.95–7.17)	0.46 (0.33–0.66)^***^	0.62 (0.43–0.90)^**^

^*^p < 0.05, ^**^p < 0.01, ^***^p < 0.001

According to the purpose of going abroad, a subgroup analysis for those who went abroad for work/migration (*n* = 462) was conducted, independent associated factors were consistent with the full sample analysis. The model showed that being aged 30–39 (adjusted HR [aHR] = 0.45, 95%CI 0.30–0.68), having advanced language proficiency (aHR = 0.30, 95%CI 0.14–0.63) and earning an annual income of $50,000: $100,000 (aHR = 0.31, 95%CI 0.20–0.50) or over $100,000 (aHR = 0.51, 95%CI = 0.33–0.81), and obtaining a nursing practice license abroad (aHR = 0.66, 95%CI = 0.44–0.99) were independently associated with a lower likelihood of nurse return to China (Table 3). Cox proportional hazards assumptions were met (Schoenfeld test *p* = 0·081).

**Table 3:** Hazard ratio of return according to timing and characteristics in work/migration subgroup

Variable^a^	No of return	Incidence rate per 100 person years (95%CI)	Unadjusted HR (95%CI)	Adjusted HR (95%CI)
Age				
29 and below	79	8.81 (7.07–10.9)	1 (reference)	1 (reference)
30–39	32	4.01 (2.8–5.68)	0.42 (0.28–0.39) ^***^	0.45 (0.30–0.68) ^**^
40 and above	15	6.88 (4.04–11.32)	0.67 (0.39–1.18)	0.70 (0.40–1.23)
Language proficiency				
Basic	32	12.08 (8.52–16.76)	1 (reference)	1 (reference)
Intermediate	84	7.37 (5.96–9.09)	0.63 (0.42–0.95) ^*^	0.87 (0.57–1.32)
Advanced	10	1.97 (1-3.71)	0.18 (0.09–0.36) ^***^	0.30 (0.14–0.63) ^**^
Average annual income abroad				
Below $50,000	70	15.15 (12.07–18.82)	1 (reference)	1 (reference)
$50,000-$100,000	28	2.99 (2.03–4.35)	0.23 (0.15–0.36) ^***^	0.31 (0.20–0.50) ^***^
Over $100,000	28	5.45 (3.72–7.87)	0.40 (0.26–0.62) ^***^	0.51 (0.33–0.81) ^**^
Whether obtain nursing practice licenses abroad				
No	34	13.68 (9.79–18.73)	1 (reference)	1 (reference)
Yes	92	5.53 (4.5–6.77)	0.49 (0.33–0.74) ^***^	0.66 (0.44–0.99) ^*^

^a^ Table 2 with p-value of adjusted HR less than 0.05 were included

^*^p < 0.05, ^**^p < 0.01, ^***^p < 0.001

## Challenges faced overseas

In the qualitative interviews, language barriers were frequently reported. Participants also highlighted the time and effort required to adapt to an English-speaking work environment:

*…need quite a long time to learn language.* (return from Singapore, 3-year abroad; No.7).

*Language was the biggest problem*. (Singapore, 8-year abroad; No.3);

Limited language proficiency often led to assignment in less critical positions, leaving nurses feeling undervalued or marginalized:

*Because of language proficiency*,* Chinese nurses are mostly not assigned to important departments; they are typically given simpler tasks.* (return from Saudi Arabia, 2-year abroad; No.2).

Qualification recognition was a significant challenge. Obtaining full legal nursing practicing licenses and completing exams was time-consuming:

*Before starting the job*,* exams*,* obtaining license*,* a lot of steps need to be done*,* and it takes a long time.* (return from USA, 1-year abroad; No.1).

Children’s education posed additional difficulties, as providing quality education abroad could be challenging:

*It’s challenging to provide good education for children abroad.* (return from USA, 1-year abroad; No.1).

Cultural differences could affect social integration and work experience. In some Muslim-majority countries, few women work outside the home, making adaptation difficult:

*The cultures are different*,* like fewer women in the Middle East will go to work.* (return from Saudi Arabia, 2-year abroad; No.2).

### Decisive reasons for return

When the results regarding *“Why did you return to China (Multiple Choice)”* were evaluated, more than half of the returning nurses (80 out of 155, 51.61%) indicated that family considerations were their primary reason for returning.

Family considerations were frequently cited by interviewees as a major factor influencing their decision to return. Several participants explained that urgent family matters, such as accidents or serious health issues, required their presence in China, which made continuing overseas work impractical:

*A family member had an accident*,* and the hospital couldn’t give me more than a month’s leave*,* so I resigned and returned.* (return from Singapore, 8-year abroad; No.3);

Beyond emergencies and practical considerations, some participants emphasized that maintaining close family ties and being reunited with loved ones remained a primary motivation for returning home:

*The main reason was all of my families were in China.* (return from Singapore, 3-year abroad; No.7).

Additionally, 26.45% (41 out of 155) returned after completing their planned education or training abroad. Assignment duration influenced return decisions, as some nurses had fixed overseas placements and intended to return once the period concluded:

*I went to the UK for a year of study*,* then returned when my time was up. I might study in other countries in the future.* (return from UK, 1-year abroad; No.4).

Improved employment conditions in China also motivated 8.39% (13 out of 155) to come back. As participants noted that rising salaries in China reduced the financial incentive to remain overseas:

*It seems that incomes in China have gone up a lot*,* making the gap with the Middle East not as big as it used to be.* (return from Saudi Arabia, 2-year abroad; No.2).

Apart from the reasons listed in the questionnaire, other motivations (28 out of 155, 18.06%) included visa expiration, the conclusion of work contracts, and the pursuit of opportunities in other countries. Permanent residency was also a key factor influencing nurses’ decisions to return. Some participants indicated that their lack of permanent residency abroad compelled them to come back:

*I haven’t gotten my American green card yet; otherwise*,* I probably wouldn’t have come back.* (return from USA, 1-year abroad; No.1).

### Challenges and opportunities after returning

For returning nurses, limited opportunities in public hospitals were a common concern. Participants reported that such hospitals often did not value international experience and that available positions were scarce:

*There are very few positions in public hospitals requiring international experience*,* and I find the workload in public hospitals too overwhelming for me.* (return from USA, 9-year abroad; No.6).

This issue was particularly evident in top-tier public hospitals, where competition was intense and labor supply abundant, leaving returnees with little advantage:

*Top-tier public hospitals in China aren’t short of nurses and don’t value international experience much. After returning*,* nurses don’t have an edge in getting into these hospitals.* (return from USA, 1-year abroad; No.1);

Another participant also noted that public hospitals impose strict age limits, and international work experience does not help to overcome this barrier:

*After returning*,* I’m too old to get into a major top-tier hospital—it’s practically impossible.* (return from Saudi Arabia, 2-year abroad; No.5).

In contrast, better opportunities in private hospitals represented a major advantage. Private hospitals were often more receptive to nurses with international experience and had workloads more comparable to overseas settings:

*Most of my peers went to private hospitals in big cities after returning.* (return from Saudi Arabia, 2-year abroad; No.2);

*Having been abroad for many years*,* I found it hard to adjust to the workload and management style in public hospitals after returning home*,* so I joined a good private hospital.* (return from Singapore, 3-year abroad; No.7).

Better workplace opportunities were also noted, as overseas experience and improved English proficiency enabled nurses to access better positions or relocate from smaller cities to more developed regions:

*My improved English proficiency led to better job opportunities*,* so I can relocate to the capital of my province. Without overseas experience*,* I might only be able to work in small cities.* (return from Saudi Arabia, 2-year abroad; No.2).

Teaching jobs were another pathway enabled by overseas experience.:

*Overseas experience opened me a teaching role in nursing after I returned.* (return from Singapore, 8-year abroad; No.3).

## Discussion

This study provides the first description of Chinese nurses returning from abroad. It reveals that nearly 30% of participants returned to China, mostly within three years. For nurses who were professionally adept had a lower likelihood of returning to China. Moreover, family consideration was the most common reason for overseas nurse returning. Additionally, returning nurses with international experience tend to find better job opportunities, preferring to work in foreign-owned private hospitals in major Chinese cities.

The study found that the nurses’ return rate was similar to Romania (32%) but higher than that observed in the Philippines (18%) [19, 20]. The relative higher return rate of Chinese nurses compared to Filipino nurses is partly due to bilateral labor agreements that help Filipinos secure long-term jobs abroad [30]. In contrast, Chinese nurses face more complex certification and exams procedures to get their nursing practice qualification abroad [31, 32], making it harder to stay, as confirmed by this study. Return rates vary by destination; Asian countries, such as Saudi Arabia and India, show higher return rates due to lower income levels; or culture similarities like those in Singapore, which make reintegration easier [33, 34]. In contrast, countries like Australia and those in the Americas present significant cultural differences, leading to “reverse culture shock” and reluctance to return [34, 35]. These patterns are consistent with other studies, underscoring that national-level factors—such as destination countries’ welfare systems, income levels, and cultural distance—shape nurses’ return migration decisions [25, 27].

With a response rate of 10.82%, the study may overestimate the return rate, but given that only about 6,500 Chinese nurses have worked abroad over the past decade compared to the 5.2 million total workforce [11, 12], the brain drain of Chinese nurses is not a major concern. Unlike nurse-exporting countries that face negative impacts from significant nurse migration [6, 36, 37], the international mobility of Chinese nurses appears limited and beneficial. Working abroad exposes them to diverse healthcare systems, enhancing China’s nursing reputation globally [32, 38]. This enhances the international prestige of Chinese nursing. Additionally, overseas experiences foster personal growth and builds professional networks between with international healthcare institutions [17, 39]. This study found that 70% of returning nurses continue in the nursing profession, bringing back advanced skills such as professionalism, multilingual abilities, and international connections. Their expertise improves nursing practices, management, research, and education, while driving innovations in services delivery [14, 22, 40]. China could benefit by developing strategies to leverage and share their international experience.

At professional level, our study found that more skilled or capable nurses—those who obtain nursing practice licenses abroad, have advanced language proficiency, and earn higher incomes—are more likely to stay abroad, a trend supported by many studies [41–43]. Notably, language barriers are a significant factor in Chinese nurses’ decisions to return, as confirmed by both the quantitative and qualitative results of this study [44–46]. As China is a non-English-speaking country, many Chinese nurses struggle with marginalization and lack of recognition, due to limited English proficiency, often requiring extended language training and adaptation to meet job demands [47, 48]. Our qualitative findings also underscored this issue, consistent with previous studies showing that such challenges may contribute to lower income, job dissatisfaction, and burnout, which in turn can motivate nurses to return to China [27].

At the personal level, the qualitative findings highlight the prominent role of family considerations, including parental health emergencies and children’s education, in nurses’ decisions to return. These results suggest that family responsibilities and traditional family-oriented values are important factors influencing return decisions [49]. Additionally, most returns occurred within the first three years, consistent with research showing that longer stays abroad reduce the likelihood of returning due to increased economic and social integration and the establishment of long-term residency [50–52]. This trend is especially evident among middle-aged migrants, many of whom have built families, social networks, and household stability overseas, making it difficult to restart their lives in China [53, 54]. Interview findings further revealed that for those who do return, reentering public hospitals poses particular challenges for middle-aged nurses, further discouraging their reintegration. Together, these professional and personal considerations help explain why some nurses choose to return while others remain abroad, highlighting the complex interplay of individual, social, and cultural factors in shaping international nurse mobility.

Due to the lack of support policies for returning nurses, returning nurses in China face challenges in securing jobs, as seen in similar studies Indonesian [55]. Public hospitals, especially top-tier ones, dominate China’s health system [56, 57], providing nearly half of the medical services despite representing 7.66% of all facilities [58]. However, returning nurses often struggle with heavy workloads, complex administration, and limited career growth, preventing them from fully leveraging their international skills [59, 60]. This mismatch making reintegration challenging. As a result, many returning nurses choose to work at foreign-funded or private hospitals, particularly in economically vibrant cities like Beijing, Shanghai, and Guangdong, consistent with previous findings [61]. Recently, wholly foreign-owned hospitals have been permitted in these regions, expanding beyond the previously allowed joint ventures [62]. This policy change is expected to drive the growth of foreign-invested hospitals and offer better job opportunities for returning nurses. To encourage innovation, public hospitals—which still provide most medical services—could create specialized roles for these nurses and hold periodic symposiums to share their global insights and expertise.

To maximize the benefits of international experience, coordinated strategies are recommended at three levels: at the government level, structured career support, streamlined licensing procedures, and formal recognition of overseas experience can reduce barriers and help improve workforce planning. In addition, establishing early communication channels with overseas nurses could facilitate their reintegration and long-term engagement; healthcare institutions, particularly public hospitals, can create specialized positions, organize professional symposia, and implement mentorship programs to facilitate adaptation and knowledge sharing; at the individual level, nurses themselves should engage in professional networks, share knowledge, and participate in domestic training to align their competencies with national healthcare standards.

### Study strengths and implications

This study is the first in China to utilize nationwide data on registered nurses who have worked abroad. By using the nurse applicant database from Credentials Verification Center for Health Professional, which encompasses a significant portion of Chinese migrant nurses, it offers a comprehensive and representative sample of this population. The combination of quantitative and qualitative methods allows for a more nuanced understanding of the return patterns, motivations, and reintegration experiences of these nurses. These findings fill a gap in knowledge on Chinese international nurses, particularly those who have returned, and can guide policies to support their reintegration into the healthcare system, helping them better apply their international experience.

### Limitations

Despite multiple attempts, the study experienced a low response rate and might introduce selection bias. Earlier applicants or nurses who had permanently settled abroad may have had outdated contact information or limited willingness to respond, potentially leading to an overestimation of the return rate, while non-respondents who were older or less educated may have been underrepresented, resulting in an underestimation of these groups. Moreover, the long study period (2009–2023) may have introduced recall bias, and policy changes such as COVID-19–related entry and exit restrictions could have influenced return outcomes. In addition, heterogeneity in respondents’ abroad timing and destinations limits subgroup comparability and interpretation. Finally, the semi-structured interview guide was not tested a priori, which may have constrained the rigor of the qualitative component; however, careful design of study instruments and analysis helped ensure that meaningful insights were still obtained.

## Conclusions

This study provides the first nationwide analysis of Chinese nurses returning from abroad. Nearly 30% of nurses returned, while those with higher skills, international licenses, advanced language proficiency, and higher incomes were more likely to stay abroad. Family considerations were the primary drivers of return decisions. After returning, many faced reintegration challenges in public hospitals and preferred private or foreign-funded hospitals in major cities. Despite the low response rate, the findings underscore opportunities to leverage returning nurses’ international experience to strengthen China’s healthcare system.

## Supplementary Information

Below is the link to the electronic supplementary material.

Supplementary Material 1: Additional file 1.docx: Online questionnaire Additional file 2.docx: Interview guide for returned nurses. Additional file 3.docx: Comparison of respondents, non-respondents, and nurses without valid email addresses. Additional file 4.docx: Distribution of abroad nurses and returning nurses.

## Data Availability

No datasets were generated or analysed during the current study.

## Abbreviations

CI: Confidence Interval
Cox: Cox Proportional Hazards Regression
HR: Hazard Ratio
aHR: Adjusted Hazard Ratio
