# Supplementary material for: The return of Chinese nurses from overseas (2009–2023): a mixed-method study on influencing factors
Source: Global Health. 2025 Nov 3;21:63. doi: 10.1186/s12992-025-01157-w (PMC12581604; doi:10.1186/s12992-025-01157-w)
Supplement: Supplementary file 1 — Supplementary Material 1: Additional file 1.docx: Online questionnaire Additional file 2.docx: Interview guide for returned nurses. Additional file 3.docx: Comparison of respondents, non-respondents, and nurses without valid email addresses. Additional file 4.docx: Distribution of abroad nurses and returning nurses. [file 12992_2025_1157_MOESM1_ESM.docx]

Additional File 1 - Online questionnaire

1. What is your name? (Optional)
2. Have you worked or resided abroad after obtaining verification of your registered nursing license in CVCHP?

- Yes
- No (Finish)

**Part one - Personal characteristics**

1. How old are you?
2. What is your highest level of education?

- Post-secondary or below
- Bachelor or above

1. What is your marital status?

- Unmarried
- Married

1. Do you have kids?

- Yes
- No

**Part two - Domestic and international working information**

1. Which province did you work in before gong abroad?
2. What kind of medical institutions did you work for before going abroad?

- Public hospital
- Private hospital
- Primary healthcare clinic
- Public health institution
- Other

1. What was your professional level in nursing practice?

- Entry-level
- Middle level
- High level

1. What was your language proficiency before going abroad?

- Basic
- Intermediate
- Advanced

1. Have you obtained qualification as a Licensed Practical Nurse in the destination country?

- Yes
- No

1. Did you work in nursing after going abroad?

- Yes
- No

1. Average annual income from working abroad (in USD):

- Below $50,000
- $50,000 - $100,000
- Over $100,000

**Part three - Abroad and return status**

1. What was the destination country you migrated to?
2. When did you go abroad (year)?
3. What was your purpose for applying for the verification and going abroad? (Multiple choice)

- Study
- Work
- Migration
- Taking the RN exam
- Other

1. What considerations contributed to you decision to go abroad?

- Salary and welfare
- Workload
- Working environment
- Personal development
- Family considerations
- Other

1. Have you returned to China?

- Yes
- No (Finish)

1. When did you return (year)?
2. Did you work in nursing after returning?

- Yes
- No (Finish)

1. Which province are you currently working in?
2. Why did you return to China? (Multiple choice)

- Dislike the living or working environment abroad
- Planned return from training or education abroad
- Improved domestic employment conditions
- Unsatisfied salaries abroad
- Workplace mistreatment
- Unable to obtain permanent residency abroad
- Language communication barriers
- Unpreventable factors (eg. public health emergency)
- Family considerations
- Other

Additional File 2-Interview Guide for Returned Nurse

1. Why did you decide to go abroad?
2. What challenges did you face in working and living overseas?

Specific probes if not answered:

-Do you find the process of obtaining nursing certification abroad complex?

-Are Chinese nurses well received in foreign medical institutions?

-What aspects did you find most difficult to adapt to while overseas?

1. What was the decisive factor that ultimately led to your decision to return to China?
2. What challenges did you face after coming back to China?

Specific probes if not answered:

-What difficulties did you find when you seek a job after returning China?

-What difficulties did you find when you work in domestic medical institutions after returning?

1. Compare to before going abroad, have you found better job opportunities in China after returning from overseas?

Specific probes if not answered:

-What skills and experiences contributed to you securing this position?

-Did your work locations differ before going abroad and after returning?

-Did your salary increase after returning from overseas compared to before going abroad?

Additional File 3 Comparison of respondents, non-respondents, and nurses without valid email addresses

Table 1 Comparison of respondents, non-respondents, and nurses without valid email addresses

| Characteristic | Respondents (n=639) | Non-respondents (valid emails)  (n=5460) | Nurses without valid email addresses (n=484) | P value |
| --- | --- | --- | --- | --- |
| Year of application  ($mean\pm SD$) | 2018.39±4.01 | 2017.16±3.72 | 2013.13±32.64 | ＜0.001^abc^ |
| Sex (*n*, *%*) |  |  |  |  |
| Female | 610 (95.46) | 5284(96.78) | 474 (97.93) | 0.064 |
| Male | 29 (4.54) | 176 (3.22) | 10 (2.66) |  |
| Age, years ($mean\pm SD$) | 37.07±7.03 | 37.66±7.41 | 40.81±7.61 | ＜0.001^ab^ |
| Ethnicity (*n*, *%*) |  |  |  |  |
| Han | 615 (96.24) | 5195 (95.15) | 457 (94.42) | 0.333 |
| Other | 24 (3.76) | 265 (4.85) | 27 (5.58) |  |
| Education (*n*, *%*) |  |  |  |  |
| Post-secondary or below | 276 (43.19) | 2948 (53.99) | 319 (65.91) | ＜0.001^abc^ |
| Bachelor or above | 363 (56.81) | 2512 (46.01) | 165 (34.09) |  |
| Level of nursing practice (*n*, *%*) |  |  |  |  |
| Entry-level | 548 (85.76) | 4821 (88.30) | 431 (89.05) | 0.110 |
| Middle level or above | 91 (14.24) | 639 (11.70) | 53 (10.95) |  |
| Language proficiency (*n*, *%*) |  |  |  |  |
| Basic | 103 (16.12) | 996 (18.24) | 87 (17.98) | 0.170 |
| Intermediate | 407 (63.89) | 3526 (64.58) | 324 (66.94) |  |
| Advanced | 129 (20.19) | 938 (17.18) | 73 (15.08) |  |

^a^ Significant difference between respondents and nurses without valid email addresses by Bonferroni.

^b^ Significant difference between non-respondents (valid emails) and nurses without valid email addresses by Bonferroni.

^c^ Significant difference between respondents and non-respondents(valid emails) by Bonferroni.

Additional File 4 - Distribution of abroad nurses and returning nurses

Table 1 - Destination countries of all nurse applicants in CVCHP (n=6583)

| Destination country | n(%) |
| --- | --- |
| United States | 3266(49.61%) |
| Australia | 1019(15.48%) |
| Singapore | 957(14.54%) |
| China (Chinese Hong Kong) | 592(8.99%) |
| Canada | 262(3.98%) |
| United Kingdom | 151(2.29%) |
| Saudi Arabia | 144(2.19%) |
| United Arab Emirates | 79(1.20%) |
| New Zealand | 74(1.12%) |
| Ireland | 15(0.23%) |
| India | 11(0.17%) |
| Finland | 4(0.06%) |
| Germany | 3(0.05%) |
| Denmark | 1(0.02%) |
| Kazakhstan | 1(0.02%) |
| Qatar | 1(0.02%) |
| Maldives | 1(0.02%) |
| Malaysia | 1(0.02%) |
| Japan | 1(0.02%) |

Table 2 - Return rates of participants by destination countries

| Destination country | Abroad nurse (n=522) | Returned nurse (n=155) | Return rate |
| --- | --- | --- | --- |
| Saudi Arabia | 20 | 20 | 100.00% |
| Singapore | 76 | 45 | 59.21% |
| India | 2 | 1 | 50.00% |
| United Arab Emirates | 2 | 1 | 50.00% |
| Hong Kong | 58 | 20 | 34.48% |
| United States of America | 207 | 49 | 23.67% |
| Ireland | 5 | 1 | 20.00% |
| United Kingdom | 21 | 4 | 19.05% |
| Canada | 23 | 3 | 13.04% |
| Australia | 99 | 10 | 10.10% |
| New Zealand | 7 | 0 | 0.00% |
| Finland | 1 | 0 | - |
| Japan | 1 | 1 | - |

Table 3 - Domestic practice locations of nurses still working on nursing after returning (n=120)

| Location | n (%) |
| --- | --- |
| Guangdong | 33 (27.50%) |
| Beijing | 21 (17.50%) |
| Shanghai | 20 (16.67%) |
| Hong Kong | 6 (5.00%) |
| Jiangsu | 5 (4.17%) |
| Zhejiang | 4 (3.33%) |
| Henan | 3 (2.50%) |
| Liaoning | 3 (2.50%) |
| Shaanxi | 3 (2.50%) |
| Gansu | 2 (1.67%) |
| Guangxi | 2 (1.67%) |
| Hubei | 2 (1.67%) |
| Hunan | 2 (1.67%) |
| Shandong | 2 (1.67%) |
| Sichuan | 2 (1.67%) |
| Chongqing | 2 (1.67%) |
| Fujian | 1 (0.83%) |
| Guizhou | 1 (0.83%) |
| Hebei | 1 (0.83%) |
| Jilin | 1 (0.83%) |
| Shanxi | 1 (0.83%) |
| Tianjin | 1 (0.83%) |
| Yunnan | 1 (0.83%) |
| Heilongjiang | 1 (0.83%) |
